# Latent TGF-beta binding protein-1 plays an important role in craniofacial development

**DOI:** 10.1590/1678-7757-2020-0262

**Published:** 2020-11-27

**Authors:** Yiting Xiong, Rongrong Sun, Jingyu Li, Yue Wu, Jingju Zhang

**Affiliations:** 1 Tongji University Shanghai Engineering Research Center of Tooth Restoration and Regeneration Department of Orthodontics, School & Hospital of Stomatology Shanghai China Tongji University, Shanghai Engineering Research Center of Tooth Restoration and Regeneration, Department of Orthodontics, School & Hospital of Stomatology, Shanghai, China.; 2 Tongji University School of Life Sciences and Technology Department of Molecular and Cell Biology Shanghai China Tongji University School of Life Sciences and Technology, Department of Molecular and Cell Biology, Shanghai, China.

**Keywords:** *LTBP1*, Craniofacial anomalies, Developmental biology, Zebrafish

## Abstract

**Objective:**

This study aims to replicate the phenotype of *Ltbp*1 knockout mice in zebrafish, and to address the function of *LTBP1* in craniofacial development.

**Methods:**

Whole mount in situ hybridization (WISH) of *ltbp1* was performed at critical periods of zebrafish craniofacial development to explore the spatial-temporal expression pattern. Furthermore, we generated morpholino based knockdown model of *ltbp1* to study the craniofacial phenotype.

**Results:**

WISH of *ltbp1* was mainly detected in the mandibular jaw region, brain trunk, and internal organs such as pancreas and gallbladder. And *ltbp1* colocalized with both *sox9a* and *ckma* in mandibular region. Morpholino based knockdown of *ltbp1* results in severe jaw malformation. Alcian blue staining revealed severe deformity of Meckel's cartilage along with the absence of ceratobranchial. Three-dimension measurements of *ltbp1* morphants jaws showed decrease in both mandible length and width and increase in open mouth distance. Expression of cartilage marker *sox9a* and muscle marker ckma was decreased in *ltbp1* morphants.

**Conclusions:**

Our experiments found that *ltbp1* was expressed in zebrafish mandibular jaw cartilages and the surrounding muscles. The *ltbp1* knockdown zebrafish exhibited phenotypes consistent with *Ltbp1* knockout mice. And loss of *ltbp1* function lead to significant mandibular jaw defects and affect both jaw cartilages and surrounding muscles.

## Introduction

The latent transforming growth factor-β binding protein-1 (*LTBP-1*) is an extracellular matrix protein that is structurally similar to the fibrillin family. There are several human and mouse *Ltbp1* splice variants including a short (*Ltbp-1S*) and long (*Ltbp-1L*) form, which may arise from the two separated promoters, alternative RNA splicing, and differential proteolytic processing.^1^ *LTBP1* is widely expressed among species in variable amounts in different tissues, including heart, lung, liver, placenta, skeletal muscle, and bone.^2^^,^^3^ Current researches suggest that *LTBP1* is a multifunctional protein that bind latent transforming growth factor-β (TGF-β) and regulate its function in bone and other connective tissues.^4^^–^^10^ *LTBP1* is reported to ease the secretion of latent TGF-β and modulate the activation of latent TGF-β.^11^^–^^16^ It is considered as a stable component of extracellular fibrillar structure that play a critical role in the storage and release of TGF-β as a large latent TGF-β complex.^17^^–^^19^ In addition to regulating the function of TGF-β, *LTBP1* function as a structural component of connective tissue microfibrils.^20^ Studies focusing on its function as matrix proteins find that *LTBP1* colocalize with fibrillin 1 in microfibrillar structures not only in the extracellular matrix of primary osteoblasts but also at the surface of newly forming osteoid and bone, suggesting that *LTBP1* play an important role in the formation of bone and connective tissue.^8^

The important function of *LTBP1* has been investigated by a series of studies. Targeted deletion of exon 1 or 2 led to *Ltbp-1L*-null mice dying shortly after birth from defects in heart organogenesis.^21^ These severe cardiac deformities include persistent truncus arteriosus and interrupted aortic arch, which are associated with imperfect function of cardiac neural crest cells. Similarly, targeted deletion of exon 8 to generate *Ltbp1L* knockout mice died perinatally, these mice also present heart defects including persistent truncus arteriosus and interrupted aortic arch which are similar to *Ltbp1L*-null mice.^22^ These studies indicate the crucial role of *LTBP1* in the development of the cardiovascular system. Another *Ltbp1* knockout mice is manipulated by targeted deletion of exon 5, which is the first exon shared by the *Ltbp1L* and *Ltbp1S*.^23^ These total knockout mice are viable and fertile, they exhibit craniofacial abnormalities consisting of more compact head structure with shorten maxilla and mandible. Furthermore, the total *Ltbp1* knockout mice have shortened long bones. Moreover, *LTBP1* knockout mice are less prone to hepatic fibrogenesis.^23^ These phenomena suggest that loss of *LTBP1* function lead to potential changes of the biological activity of TGF-β in fibrogenesis action. Researchers also used the deletion of *Ltbp1L* exon 5 knockout model to address the function of Ltbp1 in female fertility.^24^ Since *Ltbp1* knockout female mice showed impaired reproduction with subfertility and ovarian cyst formation, the interruption of TGF-β function which would lead to defective follicular wound healing was suggested as the potential cause.

It is known that *LTBP1* is critically required for the cardiovascular system organogenesis, bone and connective tissue formation, female reproduction, and craniofacial development.^21^^–^^24^ However, the discrepancy of phenotypes in *Ltbp1* gene-manipulated mice is difficult to explain. Moreover, the function of *LTBP1* in craniofacial development remains unclear. Since the gene function, fundamental signaling pathways and cellular events in craniofacial morphogenesis have proven to be highly conserved among vertebrates from zebrafish and mouse to man,^25^^,^^26^ we chose zebrafish to observe the phenotype in another model and to address the function of *LTBP1* in craniofacial aspect. We performed whole mount in situ hybridization (WISH) of *ltbp1* in critical periods of zebrafish craniofacial development to explore the spatial-temporal expression pattern. Furthermore, we generated morpholino based knockdown model of *ltbp1* to study the craniofacial phenotype.

This study aims to replicate the phenotype of *Ltbp1* knockout mice in zebrafish, and to address the function of *LTBP1* in craniofacial development.

## Methodology

### Zebrafish husbandry

Wild-type zebrafish (Tuebingen) were maintained with standard techniques.^27^ All experiments were performed in compliance with the Animal Ethics Committee.

Zebrafish embryos were collected from natural mating and kept at 28.5°C. About 300 male/female zebrafish embryos were used for detection of *ltbp1* expression pattern at five experimental periods: 1 cell, sphere, shield, 1 day postfertilization (DPF), and 3 DPF stages. About 1800 male/female zebrafish embryos were used for *ltbp1* knock-down phenotype study. The animals were divided into three experimental groups: 1. control group (wild-type zebrafish injected with control morpholino); 2. *ltbp1* knock-down group (wild-type zebrafish injected with *ltbp1 morpholino*); 3. Rescue group (wild-type zebrafish co-injected with *ltbp1* morpholino and synthesized *ltbp1* mRNA). Each group was evaluated at two experimental periods: 3 DPF and 5 DPF stages.

### Microinjection of morpholino antisense oligo and mRNA

Morpholino antisense oligos (MO) were designed to block translation and synthesis by Gene Tools, Inc. The *ltbp1*-MO was targeted to the 5′UTR of the *ltbp1* mRNA, and the *ltbp1*-MO was bound to the 5′UTR (including ATG) of the *ltbp1* mRNA. A standard control-MO was used as negative control (Table 1).^28^ Full-length cDNA was used and amplified to test knockdown specificity of the MO.^26^^,^^28^ Mutations in *ltbp1*-mRNA were introduced within the first 12 bases of open reading frame (ORF) (ATGCTgGTcTGt) without changing the sequences of amino acid so that mutated *ltbp1* mRNA could mimic *ltbp1* mRNA and it would not anneal to *ltbp1*-MO. The MOs and synthesized mRNA were injected into the yolk of embryos at the 1-cell stage. Primers used are described in Table 2. Zebrafish embryos at the 1-cell stage were injected with 1 nL of *ltbp1*-MO at a concentration ranging from 0.1 to 1 mM and a control-MO was used as a negative control. The final dosages of *ltbp1*-MO are 0.25 pmol, 0.5 pmol, and 0.75 pmol. Zebrafish embryos at the 1-cell stage were co-injected with *ltbp1*-mRNA(100 pg/embryo) and *ltbp1*-MO(0.75 pmol/embryo) for the rescue experiments.

**Table 1:** Morpholino antisense oligos

Morpholino	Sequence
*ltbp1*-MO	5′-AAACAATGATGTCCCACACGAGCAT-3
control-MO	5′-CCTCTTACCTCAGTTACAATTTATA- 3

**Table 2:** Primers for the plasmid clone

Construct	Primer	Sequence 5′-3′
*ltbp1*-mRNA	*ltbp1*-mm-ATG-EcoRI	GGAATTCcaccATGCTGGTCTGTGACATCATTGTT
	*ltbp1*-R-XhoI	CCGCTCGAGTCACTCTGTGCCCGTACTGGT

### Whole mount *in situ* hybridization

Probes for *ltbp1*, *sox9a*, and *ckma* were cloned by polymerase chain reaction with primers presented in Table 3, whole mount in situ hybridization (WISH) was performed as described.^29^ The stained embryos were photographed with a Nikon SMZ1500 stereomicroscope.

**Table 3:** Primers for the probes

probe	Primer	Sequence 5′-3′
*ltbp1*	PF-*ltbp1*-11	ATGCCAGCTCTTTGGAAGTGA
	PR-*ltbp1*-743-T7	taatacgactcactatagggGTTGATATCCACGCAGGCCA
*sox9a*	*PF-sox9a-167*	CTCCTCGACCCCTACCTGAA
	PR-*sox9a*-761-T7	taatacgactcactatagggAGATGTGGGTCTGTTCGCTG
*ckma*	PF-*ckma*-404	GTCACGGTGGATACAAGGCA
	PR-*ckma*-1246-T7	taatacgactcactatagggTCATGCTGTCGATGGACTCG

### Alcian blue staining

Embryos were fixed at 5 DPF, and cartilages were visualized by staining with alcian blue (SigmaAldrich; as described by Kimmel, et al.^30^ (1998) without using the 1.67% trypsin to digest the tissue.

### Statistical analysis

Statistical analysis was performed using GraphPad Prism 5 (GraphPad Software Inc., San Diego, CA, USA). At 5 DPF, embryos were categorized according to zebrafish jaw malformation: normal, embryos with normal cartilages compared to uninjected embryos; mild, all cartilages are generally smaller and Meckel's cartilage is mildly malformed as well as positioned ventrally, leading to mandibular jaws opened along with the defects of ceratobranchial; moderate, deformities of Meckel's cartilage are severe, jaws are widely opened and the ceratobranchial is almost completely absent; severe, increased severity of the Meckel's cartilage distortion and the ceratobranchial is completely absent; malformed, embryos with malformed general morphology. The malformed category was excluded when the percentage of cartilage was estimated. The percentage of cartilage malformation was estimated by considering the total number of mild, moderate, and severe category, and then dividing it by the total number of normal, mild, moderate, and severe category. The mean grey value of WISH staining on the mandibular cartilage (outlined by red dot line) and mandibular surrounding muscle (outlined by red dot line) was measured via ImageJ software. Measurements of lower jaw regions were conducted, including open mouth distance (Figure 3B), mandible length (Figure 3D), and mandible width (Figure 3F), the red double arrow represents the corresponding length. Each specific length was obtained by comparing the length of red double arrow with a scale bar that corresponds with the magnification of the alcian blue images.

Data are expressed as mean ± standard deviation (SD). Comparison of different groups was performed by unpaired t-test to estimate data measurements. P-value less than 0.05 (P<0.05) was considered statistically significant.

## Results

### *ltbp1* expresses at mandibular arch skeleton

Since previous studies indicated that *LTBP1* is a crucial gene in the early embryo development and it is widely expressed in several systems, we suspected that *LTBP1* was expressed in craniofacial tissues. However, the expression pattern of *LTBP1* in craniofacial tissues remains unclear. Therefore, we decided to explore the expression pattern of *LTBP1* zebrafish ortholog in craniofacial tissues.

**Figure 1:**
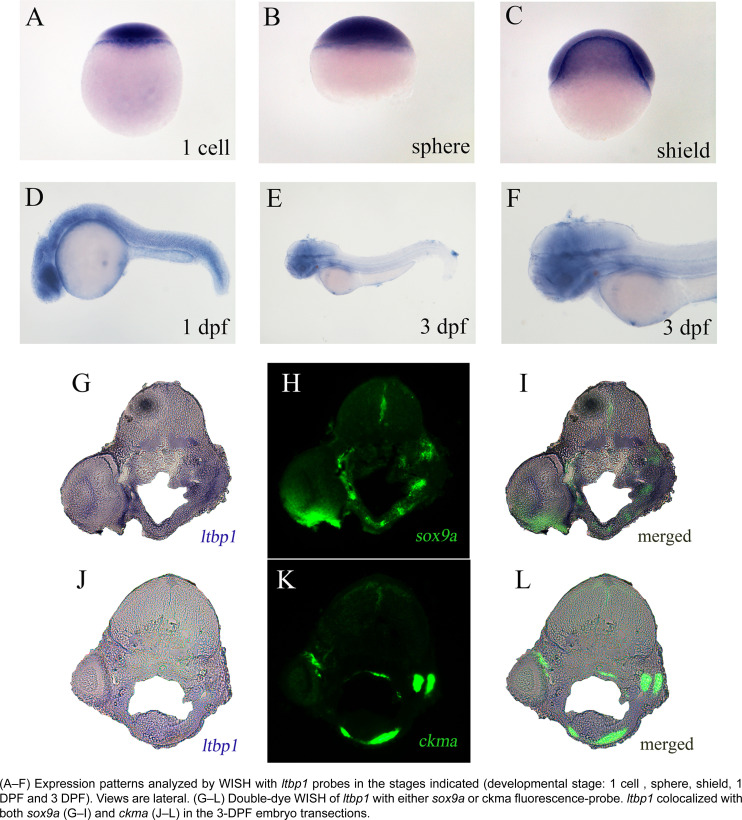
Expression patterns of *ltbp1*

According to WISH of *ltbp1* on zebrafish embryos, maternal transcripts were detected during the early developmental stage from 1 cell (Figure 1A) to shield (Figure 1C). At pharyngula period (1 DPF), *ltbp1* expressed at pharyngeal arches, brain, the most part of trunk, and the origin of internal organs (Figure 1D). In the developmental stage of protruding mouth (3 DPF), *ltbp1* was mainly detected in the mandibular jaw region and brain, but reduced expression also existed in the trunk and internal organs such as pancreas and gallbladder (Figure 1E, F). We performed 2-color WISH of *ltbp1* with cartilage marker *sox9a* and muscle marker *ckma,* respectively, to further explore the expression pattern of *ltbp1* in the mandibular jaw region. *sox9a* is a marker of cranial neural crest that differs from cartilage, and *sox9a* participates in both determination of crest-derived chondrogenic lineages and morphogenesis of cartilage (2-3 DPF)^31^. *ckma* is a terminal differentiation marker for skeletal muscle,^32^ and *ckma* was expressed in muscles of the trunk, pectoral fin, head, and heart.^33^ According to the observation of the tissue section of zebrafish jaw, *ltbp1* colocalized with both *sox9a* (Figure 1G-N) and *ckma* (Figure 1J-L). The results indicated that *ltbp1* was expressed in craniofacial tissues, and it was mainly concentrated in the zebrafish jaw cartilages and its surrounding muscles.

### Loss of *ltbp1* function Results in Severe Jaw Malformation

**Figure 2:**
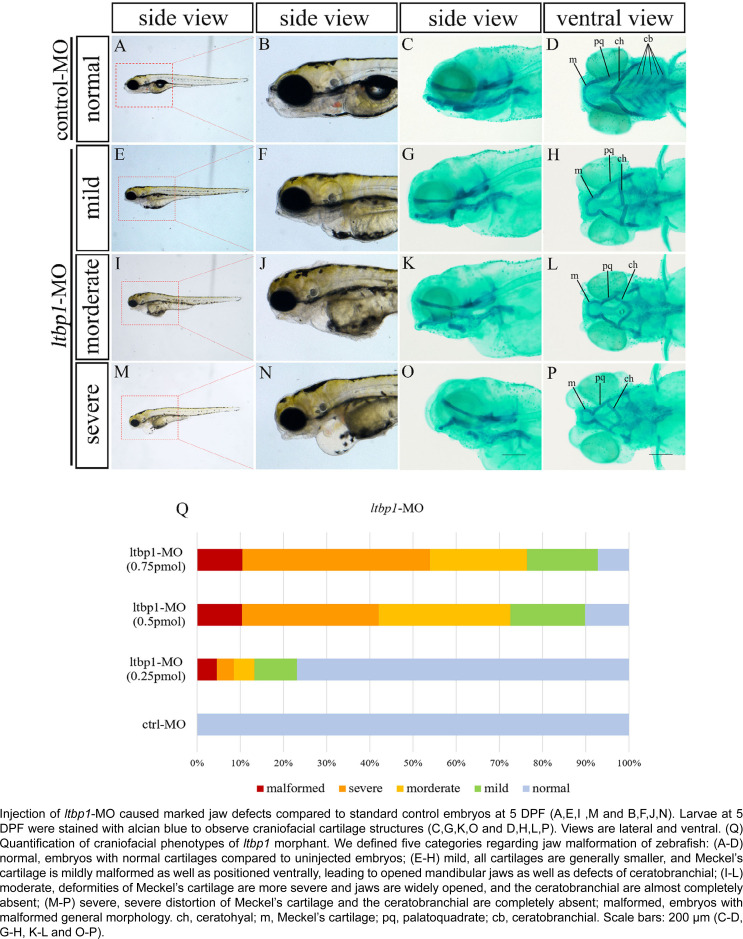
Depletion of ltbp1 causes severe jaw malformation in zebrafish

**Figure 3:**
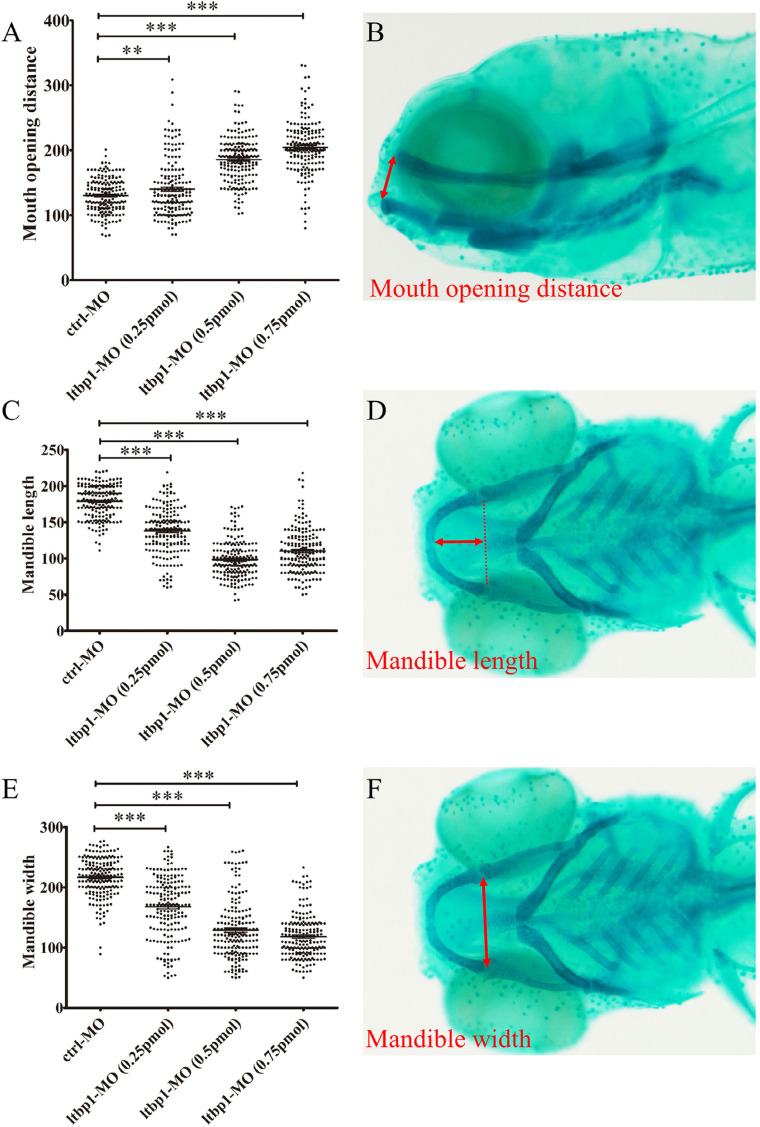
Three dimensions (3D) measurements of mandibular jaw malformation

Based on the fact that *ltbp1* is expressed in the zebrafish mandibular jaw region, we speculated that *ltbp1* play a role in the formation of mandibular jaw. The loss of *ltbp1* function was mediated by morpholino knockdown to assess the function of *ltbp1* during craniofacial development and mandibular jaw formation. We designed translation-blocking morpholino antisense oligonucleotides (MO) for *ltbp1* targeting regions in the 5’-UTR of the mRNA. MO with concentration ranging between 0.1 and 1 mM was injected 1 nL into zebrafish embryos in 1-cell stage, and a control-MO was used as a negative control. Embryos injected with control-MO were indistinguishable from uninjected embryos. Embryos injected with *ltbp1*-MO exhibited several defects, including severe jaw malformation, pericardial edema, generally shortened body length and microphthalmia. At 5 DPF, *ltbp1* morphants presented significantly underdeveloped jaw regions and the mandibular jaws were widely opened (Figure 2E-F, I-J and M-N). We performed alcian blue staining to visualize the jaw cartilage skeleton. The staining revealed severe deformity of Meckel's cartilage along with the absence of ceratobranchial (Figure 2C-D, G-H, K-L and O-P). Further measurements of lower jaw regions were conducted, including open mouth distance (Figure 3B), mandible length (Figure 3D) and mandible width (Figure 3F), in order to assess the jaw defects. The measurement of this three dimensions showed that *ltbp1* morphants decreased in both mandible length and width and it increased in open mouth distance compared to the control-MO group (Figure 3A,C,E). Interestingly, as *ltbp1* MO dosages increase, both penetrance and severity of the jaw defects increases (Figure 2Q).

**Figure 4:**
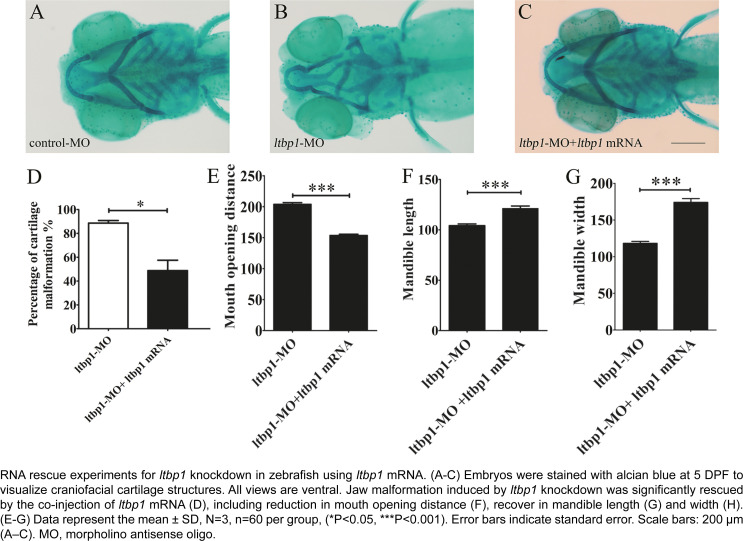
Specificity of ltbp1 knockdown phenotype

Rescue experiments with full-length *ltbp1* mRNA were carried out to confirm the specificity of *ltbp1* knockdown phenotype. Co-injection of nonhomologous *ltbp1* mRNA with *ltbp1* MO significantly rescued the jaw defects (Figure 4A-D). The rescued *ltbp1-*morphants presented increased jaw length and width, whereas their open mouth distance (Figure 4E-H) decreased, and also decreased the rescue of ceratobranchial (Figure 4C). As most phenotypes were rescued when the mRNA encoding for *ltbp1* were co-injected in the *ltbp1* MO, the off-targeting effect of *ltbp1* MO was ruled out, indicating the specificity of *ltbp1* knockdown phenotype.

### Loss of *ltbp1* function Lead to Abnormal Cartilage and Muscle during Jaw Development

**Figure 5:**
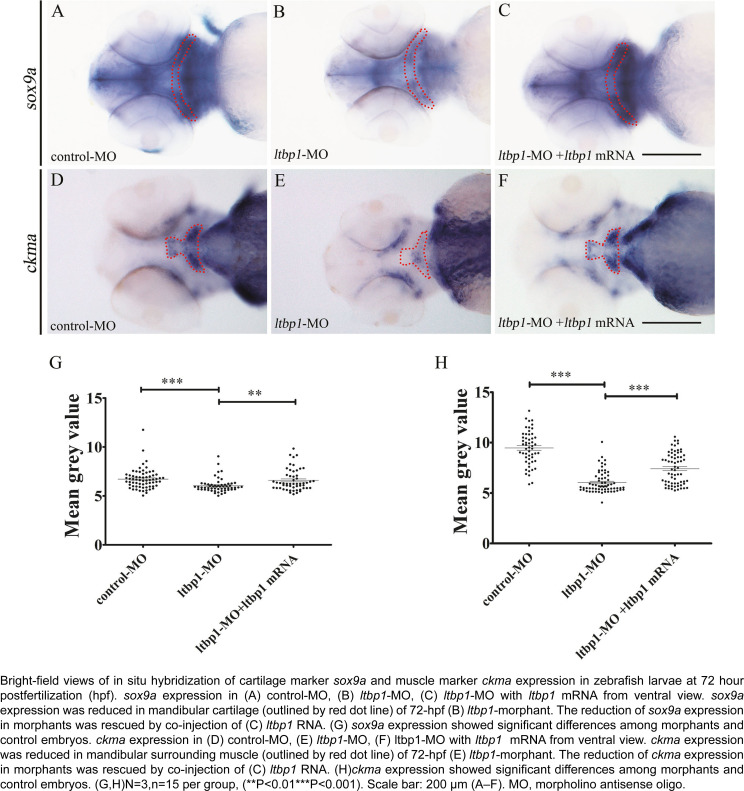
Loss of ltbp1 function affects formation of both craniofacial cartilage and muscle

Since *ltbp1* is expressed in both jaw cartilages and jaw surrounding muscles, and the loss of *ltbp1* function affected jaw development, we examined the two tissue in *ltbp1-*morphants during jaw formation. Because 3 DPF is the stage of protruding-mouth stage, when the developing cartilages of the pharyngeal skeleton such as Meckel's cartilage, has grown dramatically, we chose 3 DPF as developmental stage to examine the effects of *ltbp1* knockdown. We conducted WISH of *sox9a* and *ckma* respectively** on control-MO embryos, *ltbp1-*morphants, as well as *ltbp1* rescued morphants at 3 DPF. The expression of *sox9a* was detected in mesenchymal condensation of pharyngeal arches and jaw cartilages in control and rescued morphants, while significantly decreased in *ltbp1-*morphants (Figure 5A-C and G). Similarly, expression of *ckma* was shown in mandibular region of craniofacial muscle in control and rescued morphants, but severely reduced in *ltbp1-*morphants (Figure 5D-F and H). The WISH of *sox9a* and *ckma* indicated that *ltbp1* function loss affected jaw development in both jaw cartilage and muscle aspects.

## Discussion

*LTBP1* is an important extracellular matrix protein that regulates the bioactivity of TGF-β and also exists as microfibrillar structure of bone and connective tissue. According to recent researches, the expression of *ltbp1* is widely distributed in different tissues.^2^^,^^3^ Gene disruption experiments have shown that *LTBP1* play roles in several fields, including the development and formation of cardiovascular system, craniofacial system, reproduction system as well as functions in both bone and connective tissue.^21^^–^^24^ In this study, we explored the expression pattern of *ltbp1* using zebrafish. The results of *ltbp1* WISH showed detectable maternal transcripts from 1 cell to shield stage, which indicated that *ltbp1* is necessary during the early embryo development. In the pharyngula period (1 DPF), *ltbp1* was extensively expressed in the pharyngeal arches, brain, most part of trunk and the origin of some internal organs. In the developmental stage of protruding mouth (3 DPF), *ltbp1* expression was mainly in the mandibular jaw region and brain, while reduced expression was also present in the trunk and internal organs such as pancreas and gallbladder. The *ltbp*1 WISH results are according with previously reported widespread tissue expression. Since *ltbp1* is strongly expressed in the mandibular jaw region at 3 DPF, and the zebrafish jaw region mainly consists of cartilages and muscles. We carried out 2-colour WISH of *ltbp1* with cartilage marker *sox9a* and muscle marker *ckma* in order to specify the target tissue of *ltbp1* expression. The results showed that *ltbp1* colocalized with both *sox9a* and *ckma,* indicating that *ltbp1* concentrates in the zebrafish jaw cartilages and its surrounding muscles.

Considering that the function of a gene is usually associated with its expression pattern, we hypothesize *ltbp1* may have its function in the craniofacial development of zebrafish. According to previous studies, different strategies of *Ltbp1* knockout mice presented several phenotypes, including heart defects, craniofacial deformities, and impaired reproduction.^21^^–^^24^ However, the discrepancy of the variable phenotypes in *Ltbp1* gene-manipulated mice is complicated and ambiguous. To repeat the phenotype in another model, we performed morpholino based knockdown of *ltbp1* using zebrafish.

Generally, *ltbp1* morphants presented shortened body length, heart edema, and severe jaw malformation. At 5 DPF, *ltbp1* morphants manifested significantly underdeveloped jaw regions and the mandibular jaw was widely opened. These phenotypes are consistent with the observation of *Ltbp1* knockout mice which were built by different strategies.

In this study, we focused on jaw defects of *ltbp1* morphants, since this craniofacial aspect is the most significant observed in this phenotype, remaining unclear. Alcian blue staining of 5 DPF *ltbp1* morphants revealed severe deformity of Meckel's cartilage as well as the absence of ceratobranchial. We conducted three dimensional measurements of lower jaw regions to assess the jaw defects, and the *ltbp1* morphants decreased both mandible length and width and their open mouth distance increased. Similar phenotype was previously observed in the *Ltbp1*-null mice reported by Drews, et al.^23^ (2008), which showed modified facial profile with shortened snouts and shortened maxilla and mandible. We also found that both penetrance and severity of *ltbp1-*morphants defects of jaw increases as the dosages of *ltbp1* MO increases. Meanwhile, most phenotypes were rescued by co-injection of nonhomologous *ltbp1* mRNA with *ltbp1* MO, therefore the off-targeting effect of *ltbp1* MO was ruled out, indicating the specificity of *ltbp1* morphant phenotype. Since *ltbp1* is expressed in both jaw cartilages and surrounding muscles, we speculated that the jaw deformities could derive from the defects of either or both the two tissues. In order to address possible major aspect, we checked both cartilage and muscle markers in *ltbp1*-morphants. Both expression of *sox9a* and *ckma* was significantly decreased in *ltbp1*-morphants compared to control-MO and *ltbp1* rescued morphants, indicating that knockdown of *ltbp1* affected jaw development in both jaw cartilage and muscle aspects.

Considering that some previous studies have found that latent TGF-β is present in the matrix of chondrocytes and that *LTBP1* is responsible for storing TGF-β complex in the matrix.^6^^,^^7^ TGF-β1 and their receptors have been found to be related to zebrafish craniofacial bone and cartilage development.^34^ The cartilage development may be affected by indirect regulating function of *LTBP1* and TGF-β1. Besides, *LTBP1* may directly affect chondrocytes as an important extracellular matrix protein. Furthermore, *LTBP1* may participate in the muscle formation as microfibrillar structures of connective tissue^8^. Future studies will investigate the biological activity of TGF-β and extracellular fibrillar structure whether they are compromised or not. Although the specific mechanisms require further research, our results have extended the understanding of the important role of *LTBP1* especially in craniofacial development, easing future investigations on the mechanisms of *LTBP1* gene function.

## Conclusion

Our experiments found that *ltbp1* is expressed in zebrafish mandibular jaw cartilages and the surrounding muscles in addition to the previously reported tissues. Also, the *ltbp1* knockdown zebrafish presented phenotypes consistent with *Ltbp1* knockout mice. Lastly, loss of *ltbp1* function lead to significant mandibular jaw defects and affect both jaw cartilages and surrounding muscles.

## References

[1] 1- Koski C, Saharinen J, Keski-Oja J. Independent promoters regulate the expression of two amino terminally distinct forms of latent transforming growth factor-beta binding protein-1 (*LTBP-1*) in a cell type-specific manner. J Biol Chem. 1999;274(46):32619-30. doi:10.1074/jbc.274.46.32619. 10.1074/jbc.274.46.32619 10551816

[2] 2- Weiskirchen R, Moser M, Günther K, Weiskirchen S, Gressner AM. The murine latent transforming growth factor-beta binding protein (*Ltbp-1*) is alternatively spliced, and maps to a region syntenic to human chromosome 2p21-22. Gene. 2003;308:43-52. doi:10.1016/s0378-1119(03)00464-5 10.1016/s0378-1119(03)00464-5 12711389

[3] 3- Faraoni EY, Camilletti MA, Abeledo-Machado A, Laura DR, Ratner F, De Fino F, et al. Sex differences in the development of prolactinoma in mice overexpressing hCGβ: role of TGFβ1. J Endocrinol. 2017;232(3):535-46. doi:10.1530/JOE-16-0371 10.1530/JOE-16-0371 28096433

[4] 4- Akhurst RJ, Lehnert SA, Faissner A, Duffie E. TGF beta in murine morphogenetic processes: the early embryo and cardiogenesis. Development. 1990;108(4):645-56. 10.1242/dev.108.4.645 1696875

[5] 5- Nakajima Y, Miyazono K, Nakamura H. Immunolocalization of latent transforming growth factor-beta binding protein-1 (*LTBP1*) during mouse development: possible roles in epithelial and mesenchymal cytodifferentiation. Cell Tissue Res. 1999;295(2):257-67. doi:10.1007/s004410051232 10.1007/s004410051232 9931372

[6] 6- Pedrozo HA, Schwartz Z, Gomez R, Ornoy A, Xin-Sheng W, Dallas SL, et al. Growth plate chondrocytes store latent transforming growth factor (TGF)-beta 1 in their matrix through latent TGF-beta 1 binding protein-1. J Cell Physiol. 1998;177(2):343-54. doi:10.1002/(SICI)1097-4652(199811)177:2<343::AID-JCP16>3.0.CO;2-A 10.1002/(SICI)1097-4652(199811)177:2<343::AID-JCP16>3.0.CO;2-A 9766531

[7] 7- Pedrozo HA, Schwartz Z, Mokeyev T, Ornoy A, Xin-Sheng W, Bonewald LF, et al. Vitamin D3 metabolites regulate LTBP1 and latent TGF-beta1 expression and latent TGF-beta1 incorporation in the extracellular matrix of chondrocytes. J Cell Biochem. 1999;72(1):151-65. 10025676

[8] 8- Dallas SL, Keene DR, Bruder SP, Saharinen J, Sakai LY, Mundy GR, et al. Role of the latent transforming growth factor beta binding protein 1 in fibrillin-containing microfibrils in bone cells *in vitro* and *in vivo*. J Bone Miner Res. 2000;15(1):68-81. doi:10.1359/jbmr.2000.15.1.68 10.1359/jbmr.2000.15.1.68 10646116

[9] 9- Ota T, Fujii M, Sugizaki T, Ishii M, Miyazawa K, Aburatani H, et al. Targets of transcriptional regulation by two distinct type I receptors for transforming growth factor-beta in human umbilical vein endothelial cells. J Cell Physiol. 2002;193(3):299-318. doi:10.1002/jcp.10170 10.1002/jcp.10170 12384983

[10] 10- Vittal R, Mickler EA, Fisher AJ, Zhang C, Rothhaar K, Gu H, et al. Type V collagen induced tolerance suppresses collagen deposition, TGF-β and associated transcripts in pulmonary fibrosis [published correction appears in PLoS One. 2018;13(12):e0209107]. PLoS One. 2013;8(10):e76451. Published 2013 Oct 21. doi:10.1371/journal.pone.0076451 10.1371/journal.pone.0209107 PMC6283573 30521637

[11] 11- Nakajima Y, Miyazono K, Kato M, Takase M, Yamagishi T, Nakamura H. Extracellular fibrillar structure of latent TGF beta binding protein-1: role in TGF beta-dependent endothelial-mesenchymal transformation during endocardial cushion tissue formation in mouse embryonic heart. J Cell Biol. 1997;136(1):193-204. doi:10.1083/jcb.136.1.193 10.1083/jcb.136.1.193 PMC2132455 9008713

[12] 12- Nakano M, Arai E, Nakajima Y, Nakamura H, Miyazono K, Hirose T. Immunohistochemical study of chondrolipoma: possible importance of transforming growth factor (TGF)-betas, latent TGF-beta binding protein-1 (*LTBP-1*), and bone morphogenetic protein (BMP) for chondrogenesis in lipoma. J Dermatol. 2003;30(3):189-95. doi:10.1111/j.1346-8138.2003.tb00370.x 10.1111/j.1346-8138.2003.tb00370.x 12692354

[13] 13- Gui Y, Murphy LJ. Interaction of insulin-like growth factor binding protein-3 with latent transforming growth factor-beta binding protein-1. Mol Cell Biochem. 2003;250(1-2):189-95. doi:10.1023/a:1024990409102 10.1023/a:1024990409102 12962157

[14] 14- Ge G, Greenspan DS. BMP1 controls TGF beta1 activation via cleavage of latent TGFbeta-binding protein. J Cell Biol. 2006;175(1):111-20. doi:10.1083/jcb.200606058 10.1083/jcb.200606058 PMC2064503 17015622

[15] 15- Maurya VK, Jha RK, Kumar V, Joshi A, Chadchan S, Mohane JJ, et al. Transforming growth factor-beta 1 (TGF-B1) liberation from its latent complex during embryo implantation and its regulation by estradiol in mouse [published correction appears in Biol Reprod. 2014;91(6):147]. Biol Reprod. 2013;89(4):84. Published 2013 Oct 10. doi:10.1095/biolreprod.112.106542 10.1095/biolreprod.112.106542 23926286

[16] 16- Zhao Q, Zheng K, Ma C, Li J, Zhuo L, Huang W, et al. PTPS facilitates compartmentalized *LTBP1* S-nitrosylation and promotes tumor growth under hypoxia. Mol Cell. 2020;77(1):95-107.e5. doi:10.1016/j.molcel.2019.09.018 10.1016/j.molcel.2019.09.018 31628042

[17] 17- Robertson IB, Handford PA, Redfield C. Backbone ^1^H, ^13^C and ^15^N resonance assignment of the C-terminal EGF-cbEGF pair of *LTBP1* and flanking residues. Biomol NMR Assign. 2014;8(1):159-63. doi:10.1007/s12104-013-9474-6. 10.1007/s12104-013-9474-6 23494870

[18] 18- Robertson IB, Handford PA, Redfield C. NMR spectroscopic and bioinformatic analyses of the *LTBP1* C-terminus reveal a highly dynamic domain organisation. PLoS One. 2014;9(1):e87125. doi:10.1371/journal.pone.0087125 10.1371/journal.pone.0087125 PMC3906135 24489852

[19] 19- Chen H, Cai W, Chu ES, Tang J, Wong CC, Wong SH, et al. Hepatic cyclooxygenase-2 overexpression induced spontaneous hepatocellular carcinoma formation in mice. Oncogene. 2017;36(31):4415-26. doi:10.1038/onc.2017.73 10.1038/onc.2017.73 PMC5543258 28346420

[20] 20- Hubmacher D, Wang LW, Mecham RP, Reinhardt DP, Apte SS. Adamtsl2 deletion results in bronchial fibrillin microfibril accumulation and bronchial epithelial dysplasia--a novel mouse model providing insights into geleophysic dysplasia. Dis Model Mech. 2015;8(5):487-99. doi:10.1242/dmm.017046 10.1242/dmm.017046 PMC4415891 25762570

[21] 21- Todorovic V, Frendewey D, Gutstein DE, Chen Y, Freyer L, Finnegan E, et al. Long form of latent TGF-beta binding protein 1 (*Ltbp1L*) is essential for cardiac outflow tract septation and remodeling. Development. 2007;134(20):3723-32. doi:10.1242/dev.008599 10.1242/dev.008599 17804598

[22] 22- Horiguchi M, Todorovic V, Hadjiolova K, Weiskirchen R, Rifkin DB. Abrogation of both short and long forms of latent transforming growth factor-β binding protein-1 causes defective cardiovascular development and is perinatally lethal. Matrix Biol. 2015;43:61-70. doi:10.1016/j.matbio.2015.03.006 10.1016/j.matbio.2015.03.006 PMC4547347 25805620

[23] 23- Drews F, Knöbel S, Moser M, Muhlack KG, Mohren S, Stoll C, et al. Disruption of the latent transforming growth factor-beta binding protein-1 gene causes alteration in facial structure and influences TGF-beta bioavailability. Biochim Biophys Acta. 2008;1783(1):34-48. doi:10.1016/j.bbamcr.2007.08.004 10.1016/j.bbamcr.2007.08.004 17950478

[24] 24- Dietzel E, Weiskirchen S, Floehr J, Horiguchi M, Todorovic V, Rifkin DB, et al. Latent TGF-β binding protein-1 deficiency decreases female fertility. Biochem Biophys Res Commun. 2017;482(4):1387-92. doi:10.1016/j.bbrc.2016.12.046 10.1016/j.bbrc.2016.12.046 27956181

[25] 25- Mork L, Crump G. Zebrafish craniofacial development: a window into early patterning. Curr Top Dev Biol. 2015;115:235-269. doi:10.1016/bs.ctdb.2015.07.001 10.1016/bs.ctdb.2015.07.001 PMC4758817 26589928

[26] 26- Machado RG, Eames BF. Using zebrafish to test the genetic basis of human craniofacial diseases. J Dent Res. 2017;96(11):1192-9. doi:10.1177/0022034517722776 10.1177/0022034517722776 28767277

[27] 27- Kimmel CB, Ballard WW, Kimmel SR, Ullmann B, Schilling TF. Stages of embryonic development of the zebrafish. Dev Dyn. 1995;203(3):253-310. doi:10.1002/aja.1002030302 10.1002/aja.1002030302 8589427

[28] 28- Stainier DY, Raz E, Lawson ND, Ekker SC, Burdine RD, Eisene JS, et al. Guidelines for morpholino use in zebrafish. PLoS Genet. 2017;13(10):e1007000. doi:10.1371/journal.pgen.1007000 10.1371/journal.pgen.1007000 PMC5648102 29049395

[29] 29- Thisse B, Thisse C. *In situ* hybridization on whole-mount zebrafish embryos and young larvae. Methods Mol Biol. 2014;1211:53-67. doi:10.1007/978-1-4939-1459-3_5 10.1007/978-1-4939-1459-3_5 25218376

[30] 30- Kimmel CB, Miller CT, Kruze G, Ullmann B, BreMiller RA, Larison KD, et al. The shaping of pharyngeal cartilages during early development of the zebrafish. Dev Biol. 1998;203(2):245-63. doi:10.1006/dbio.1998.9016 10.1006/dbio.1998.9016 9808777

[31] 31- Yan YL, Willoughby J, Liu D, Crump JG, Wilson C, Miller CT, et al. A pair of sox: distinct and overlapping functions of zebrafish *sox9* co-orthologs in craniofacial and pectoral fin development. Development. 2005;132(5):1069-83. doi:10.1242/dev.01674 10.1242/dev.01674 15689370

[32] 32- Xu Y, He J, Wang X, Lim TM, Gong Z. Asynchronous activation of 10 muscle-specific protein (MSP) genes during zebrafish somitogenesis. Dev Dyn. 2000;219(2):201-15. doi:10.1002/1097-0177(2000)9999:9999<::aid-dvdy1043>3.3.co;2-9 10.1002/1097-0177(2000)9999:9999<::aid-dvdy1043>3.3.co;2-9 11002340

[33] 33- Thisse B, Pflumio S, Fürthauer M, Loppin B, Heyer V, Degrave A, et al. Expression of the zebrafish genome during embryogenesis (NIH R01 RR15402) [Internet]. Eugene: ZFIN; 2001 [cited 2020 Aug 5]. Available from: https://zfin.org/ZDB-PUB-010810-1

[34] 34- Zhang Y, Ji D, Li L, Yang S, Zhang H, Duan X. ClC-7 Regulates the pattern and early development of craniofacial bone and tooth. Theranostics. 2019;9(5):1387-400. doi:10.7150/thno.29761 10.7150/thno.29761 PMC6401512 30867839

